# Varying concentrations of tyrosine kinase inhibitors in chronic myeloid leukemia patients following bariatric surgery: a case series

**DOI:** 10.1007/s00277-024-05924-4

**Published:** 2024-08-12

**Authors:** Cedric Lau, Charlotte van Kesteren, Yong Xin Cao, Robert M. Smeenk, Laura G.M. Daenen, Jeroen J.W.M. Janssen, Peter E. Westerweel

**Affiliations:** 1 Department of Clinical Pharmacy, Albert Schweitzer Hospital, Albert Schweitzerplaats 25, Dordrecht, 3318 AT The Netherlands; 2 Department of Pharmacy and Pharmacology, The Netherlands Cancer Institute, Plesmanlaan 121, Amsterdam, 1066 CX The Netherlands; 3 Division of Pharmacology, The Netherlands Cancer Institute, Plesmanlaan 121, Amsterdam, 1066 CX The Netherlands; 4 Department of Clinical Pharmacy, University Medical Center Utrecht, Utrecht University, Heidelberglaan 100, Utrecht, 3584 CX The Netherlands; 5 Department of Surgery, Albert Schweitzer Hospital, Albert Schweitzerplaats 25, Dordrecht, 3318 AT The Netherlands; 6 Department of Hematology, University Medical Center Utrecht, Utrecht University, Heidelberglaan 100, Utrecht, 3584 CX The Netherlands; 7 Department of Hematology, Radboud University Medical Center, Geert Grooteplein Zuid 10, Nijmegen, 6525 GA The Netherlands; 8 Department of Internal Medicine, Albert Schweitzer Hospital, Albert Schweitzerplaats 25, Dordrecht, 3318 AT The Netherlands

**Keywords:** Bariatric surgery, Roux-en-Y gastric bypass, Sleeve gastrectomy, Chronic myeloid leukemia, Tyrosine kinase inhibitors, Pharmacokinetics, Therapeutic drug monitoring

## Abstract

Bariatric surgery is increasingly performed to treat severe obesity. As a result of anatomical and physiological changes in the gastrointestinal tract, the pharmacokinetics (PK) of oral drugs can be altered, affecting their efficacy and safety. This includes the class of tyrosine kinase inhibitors (TKIs) which are used to treat chronic myeloid leukemia (CML). This case series describes the clinical course of four CML cases with a history of bariatric surgery. The patients used various TKIs (nilotinib, dasatinib, bosutinib, ponatinib, and imatinib) for which 15 drug levels were measured. The measured TKI concentrations were in part subtherapeutic, and highly variable when compared to mean levels measured in the general population. Multiple drug levels were measured in these patients, as the clinicians were aware of the possible impact of bariatric surgery. The drug levels were used as additional input for clinical decision-making. All four patients required TKI switches and/or dose modifications to achieve an effective and tolerable treatment. Eventually, adequate clinical and molecular remissions were achieved in all cases. In summary, TKI concentrations of patients undergoing bariatric surgery may be subtherapeutic. Moreover, there is substantial interindividual and intraindividual variation, which may be explained by the complex interference of bariatric surgery and associated weight loss. For clinical practice, therapeutic drug monitoring is advised in patients with a history of bariatric surgery in case of suboptimal response or loss of response.

## Introduction

The performance of bariatric surgery as a treatment modality for obesity has strongly increased over the years [1]. Currently, the most performed procedures are *sleeve gastrectomy* (SG) and *Roux-en-Y gastric bypass* (RYGB) [1]. RYGB is a surgical procedure that involves decreasing the size of the stomach along with attaching the small-sized stomach directly to the Roux limb. With SG, a part along the greater curvature of the stomach is removed, reducing its size to about 15% of the original stomach. These surgical treatments lead to restricted food intake and reduced absorption of enteral nutrients [2].

The bariatric surgical procedures result in physiological changes in the gastrointestinal tract and substantial weight loss. These changes can lead to altered absorption, distribution, metabolism, and elimination of drugs, and thus can significantly impact the pharmacokinetics (PK) of drugs [3]. After both SG and RYGB, patients have a shorter gastric emptying time [4] and a higher pH [5, 6]. In SG patients, the gastric pH returns to preoperative values after one year [5]. Furthermore, the reduction in gastric volume in both types of surgery could interfere with the disintegration and dissolution of oral drugs [7, 8]. A higher pH results in improved solubility of acidic drugs, while the solubility of basic drugs might be impaired. An example is the malabsorption of the basic drug tamoxifen in RYGB patients, as tamoxifen [9] and endoxifen [10] levels were below the therapeutic range after the surgery.

The altered pharmacokinetics induced by the aforementioned physiological changes imply that dose adjustments following bariatric surgery may be necessary to achieve effective blood levels. Currently, there is limited data on the effects of bariatric surgery on most drugs [11–14], including the BCR::ABL tyrosine kinase inhibitors (TKIs). These drugs form the cornerstone of chronic myeloid leukemia (CML) treatment with an excellent prognosis for the majority of patients, but mostly requiring lifelong use. Currently available TKIs include imatinib, dasatinib, nilotinib, bosutinib, ponatinib, and asciminib [15].

Various studies have shown a relationship between the exposure to TKIs and the effect of the treatment [16–18]. Based on these studies, the minimum target levels considered to be required to achieve the optimal effect of these drugs are trough level > 1000 ng/mL for imatinib [19], trough level ≥ 800 ng/mL for nilotinib [19, 20] and peak level > 50 ng/mL for dasatinib [19]. For dasatinib, the trough level is related to toxicity (pleural effusion) rather than efficacy. The recommended dasatinib trough level is ≤ 2.5–2.61 ng/mL [19, 21, 22].

Less evidence on minimal target levels is available for bosutinib and ponatinib, although details on the trough level found in the general population are available for reference. Studies found the trough level of bosutinib to be 75–215 ng/mL (median 147 ng/mL) and ponatinib to be ≥ 34 ng/mL (at a dose of 45 mg OD) [21].

A recent study demonstrated that bariatric surgery is associated with an inferior overall survival (OS) in TKI-treated CML patients [23]. Responses to TKIs were slower and the rate of deep remission was lower in patients with previous bariatric surgery. However, in the available study, plasma TKI levels were not measured [23].

Thus, the effect of bariatric surgery on the absorption and efficacy of the TKI remains speculative. Measuring TKI blood levels in CML patients who have previously undergone bariatric surgery is essential to understand if altered pharmacokinetics occur that may undermine responses to TKI treatment in standard dosages. We present a case series reporting four CML patients with measured TKI drug levels before and after bariatric surgery.

## Case descriptions

### Case 1

A Caucasian woman, diagnosed with CML at 30 years old, presented with treatment failure to several lines of TKI treatment. The patient had a history of class II obesity and had undergone RYGB in September 2017 at a weight of 96.5 kg pre-surgery corresponding to a BMI of 36.1 kg/m^2^. Six months later, in March 2018, she was diagnosed with CML and she was started on imatinib at a standard dose of 400 mg QD. One month later, the patient proved intolerant to imatinib due to nausea as an adverse event (AE) and consequently switched to nilotinib 400 mg BID. Concomitantly, she was prescribed omeprazole 40 mg QD in September 2018 to alleviate pyrosis. By this time, her weight had been reduced to 57 kg (BMI 21.5 kg/m^2^).

With nilotinib treatment, she initially responded well to therapy and achieved a Major Molecular Response (MMR) in 2019 with a BCR::ABL < 0.1% on the International Scale (IS). Her BCR::ABL remained below 0.1% and even went to ≤ 0.001% corresponding with an MR5 in the first half of 2020. Because of AEs including myalgia, stomach aches, palpitations, and cold feet, the nilotinib dose was reduced to 300 mg BID. The nilotinib trough level at a 300 mg BID regimen was 304 ng/mL (Table 1), which was far below the target threshold value for therapeutic effect (≥ 800 ng/mL). Due to the AEs, she was switched to dasatinib 100 mg QD. She again experienced gastrointestinal AEs (diarrhea and vomiting), headache, myalgia, and arthralgia. Because of this intolerance, the dose of dasatinib was lowered to 70 mg QD and eventually 50 mg QD. No plasma levels of dasatinib were measured at these doses. Initially, the BCR::ABL remained stably < 0.1% IS. However, 7 months later, the patient lost the MMR. She discontinued omeprazole to rule out the use of the proton pump inhibitor as the cause of the subtherapeutic TKI effect, but this attempt was to no avail. There was an increase of the BCR::ABL to > 1% IS, after which dasatinib dosages were increased up to 180 mg QD. During this time, four dasatinib trough levels were measured at different doses. At 70 mg, 100 mg, 140 mg, and 180 mg QD, trough levels were measured at respectively 2.6 ng/mL, 1.57 ng/mL, 1.54 ng/mL, and 1.83 ng/mL (reference ≤ 2.5 ng/mL). No peak levels were measured for this patient, which would have been clinically more relevant to assess efficacy.

**Table 1:** Overview of used TKI and corresponding doses and plasma or serum levels if available

Case	Type of surgery	Drug	Time after surgery	Dose (mg)	Measured drug concentration	Target drug concentration	Interpretation of concentration
1	RYGB	Imatinib	6 m	400 QD	N/A	> 1000 µg/L	N/A
		Nilotinib	7 m	400 BID	N/A	≥ 800 µg/L	N/A
			1 y 4 m	300 BID	304 ng/mL		Subtherapeutic
		Dasatinib	1 y 7 m	100 QD	N/A	Trough < 2.5 ng/mL Peak ≥ 50 ng/mL	N/A
			1 y 9 m	70 QD	N/A		
			2 y	50 QD			
			2 y 7 m	70 QD	2.6 ng/mL (trough)		Trough level minimally beyond the toxic threshold
			2 y 11 m	100 QD	1.57 ng/mL (trough)		Trough level below the toxic threshold
			3 y 2 m	140 QD	1.54 ng/mL (trough)		
			3 y 4 m	180 QD	1.83 ng/mL (trough)		
		Nilotinib	3 y 6 m	300 BID	560 ng/mL	≥ 800 µg/L	Subtherapeutic
			3 y 7 m	400 BID	1060 ng/mL		Therapeutic
			3 y 10 m	600 BID	542 ng/mL		Subtherapeutic
		Ponatinib	4 y	45 QD	N/A	No target. Median level 34.2 µg/L at dose of 45 mg OD	N/A
			4 y	30 QD			
			4 y 4 m	15 QD	11 µg/L*		As expected
2	SG	Nilotinib	2 m	150–450	728 ng/mL	≥ 800 µg/L	Subtherapeutic
			2 y	300 BID	N/A		N/A
			2 y 7 m	200 BID			
			2 y 10 m	150 BID			
		Bosutinib	3 y 8 m	200 QD	N/A	No target. Dose range in the general population: 75–215 µg/L	N/A
			3 y 9 m	300 QD			
			3 y 10 m	400 QD			
			3 y 11 m	300 QD			
			4 y 1 m	200 QD			
			5 y 6 m	300 QD			
			6 y 6 m	400 QD	46 µg/L*		Subtherapeutic
3	RYGB	Nilotinib	Before	300 BID	801 µg/L	≥ 800 µg/L	Therapeutic
		Bosutinib	9 m	400 QD	32.5 µg/L	No target. Dose range in the general population: 75–215 µg/L	Subtherapeutic
4	RYGB	Dasatinib	Before	100 QD	N/A	Trough < 2.5 ng/mL Peak ≥ 50 ng/mL	N/A
		Imatinib	7 m	400 QD	586 µg/L	> 1000 µg/L	Subtherapeutic
			1 y 4 m	400 QD	814 µg/L	> 1000 µg/L	Subtherapeutic

BID = twice daily, m = months, N/A = not available, QD = once daily, RYGB = Roux-en-Y gastric bypass, SG = sleeve gastrectomy, y = years, * = after log-linear extrapolation

The BCR::ABL continued to rise, leading the patient to switch back to nilotinib. During 2021, she used 300 mg BID up to 600 mg BID nilotinib. However, the increases in dose did not lead to a desired BCR::ABL, while the patient suffered from AEs (myalgia, arthralgia, and rash). Nilotinib trough levels were measured at different dates. In chronological order, the first trough level was too low (560 ng/mL at 300 mg BID), the second was adequate (1060 ng/mL at 400 mg BID), and the third was too low as well (542 ng/mL at 600 mg BID).

Due to the lack of therapeutic response, ponatinib 45 mg QD monotherapy was started in September 2021. AEs (headache, muscle ache, skin rash, increased blood pressure) occurred, which diminished after reducing the dose to 30 mg QD. As MMR was reached, the dose of ponatinib was reduced to 15 mg QD, which she still uses at the last follow-up in March 2024. Some side effects persist at a mild to moderate level (headache, myalgia, skin rash, and fatigue). The measured trough concentration was 15 µg/L. By means of log-linear extrapolation using the median elimination rate constant as previously reported by Wang et al. [24], the expected ponatinib trough concentration for this patient was 11 µg/L.

### Case 2

A Black woman, diagnosed with CML at 35 years of age, was started on imatinib in 1998 through a clinical trial, after which she was in stable remission. She underwent gastric banding in 2007, which was removed shortly after due to surgical complications. At this time in 2007, she switched to dasatinib due to AEs on imatinib. Eventually, a subsequent switch to nilotinib was done in 2014 because of AEs on dasatinib and a suboptimal response. After the start of nilotinib, the patient underwent an SG surgery after which she lost 15 kg in 2 months. Following SG surgery, in October 2015, a nilotinib trough level was measured at 728 ng/mL (Table 1), which indicates that the concentration was subtherapeutic (reference ≥ 800 µg/L). Her CML remained stable, as BCR::ABL has been measured within the ranges 0.111–0.33% (2017), 0.097–0.21% (2018), and 0.1–0.2% IS (2019).

Nilotinib was used until mid-2019. The patient experienced various AEs under nilotinib resulting in multiple changes in the regimen including discontinuation with rechallenge and trial dose reductions. In response to intolerance of nilotinib at standard doses and loss of MMR at lower doses, the patient was switched to bosutinib 400 mg QD after a ramp-up period of 200 mg QD and then 300 mg QD. At 400 mg QD, the patient experienced clinically significant headaches for which the dose of bosutinib was temporarily reduced to 300 mg QD and later 200 mg QD. However, the dose was later successfully increased again to 400 mg QD with acceptable tolerability and fluctuating BCR::ABL levels between 0.1 and 1% IS. The measured bosutinib concentration was 52 µg/L. After log-linear extrapolation, the trough concentration would be 46 µg/L (reference 75–215 µg/L). Currently, this dose is reasonably well tolerated, and the patient is again in MMR.

### Case 3

A 53-year-old Caucasian woman, diagnosed with CML at 46 years of age, underwent a RYGB in September 2021. Before the RYGB, this patient was stable on nilotinib with adequate BCR::ABL responses around 0.01% IS. Several years before surgery, the concentration of nilotinib was 801 µg/L (reference at that moment 500–1500 µg/L). Following surgery, she received bosutinib 400 mg QD (Table 1). Her BCR::ABL in June 2022 was 0.16% along with a measured bosutinib trough concentration of 32.5 µg/L (reference 75–215 µg/L). Her weight was 78 kg. She used omeprazole 20 mg QD concomitantly.

### Case 4

A 57-year-old Caucasian man, diagnosed with CML at 47 years of age in 2012, initially received imatinib 400 mg QD at first diagnosis. For a deeper response, he switched to dasatinib 100 mg QD in 2014. Due to relapsing pleural fluid, he switched back to imatinib 400 mg QD in 2017, maintaining MMR. Before bariatric surgery, his response was an MR5 and his weight was 125 kg (BMI 36 kg/m^2^). At 7 months after RYGB (October 2022), his imatinib trough concentration was 586 µg/L (reference > 1000 µg/L) and his weight had reduced to 89 kg (BMI 26 kg/m^2^). At 16 months after RYGB, his imatinib trough concentration was 814 µg/L (Table 1) and his weight had reduced to 80 kg (BMI 23 kg/m^2^). Despite these low trough concentrations, he remained in MR5. He did not use any other medication except for hydrochlorothiazide.

## Discussion

To achieve an optimal therapeutic response and minimize potentially avoidable AEs, it is crucial to maintain adequate TKI concentrations within the appropriate therapeutic range [16, 21, 25, 26]. Since CML patients with previous bariatric surgery have an inferior prognosis [23], we aimed to describe four CML patients with bariatric surgery and the measured drug concentrations of TKI.

In these patients, clinical challenges were prominent. On the one hand, various AEs occurred, in part clinically suggested to be dose-dependent. On the other hand, treatment responses were suboptimal at multiple time points. Together, this necessitated multiple changes in TKI type and dose. Multiple drug level measurements were performed in these patients as the clinicians were aware of the possible impact of bariatric surgery. Results were used as additional input for clinical decision-making. Eventually, adequate clinical and molecular remissions were achieved in all four cases. This is an important result in these patients, as a previous report documented inferior treatment response and failure-free survival in CML patients with a history of bariatric surgery [23].

Measuring drug concentrations or *Therapeutic Drug Monitoring* (TDM) in patients with bariatric surgery may be clinically useful. In this case series, several TKI concentrations were subtherapeutic. The imatinib levels in case 4 were below the therapeutic range, in line with two earlier case reports [27, 28], which are the only currently published observations of TKI concentrations in CML patients after bariatric surgery. Both these cases demonstrated lower trough levels of imatinib [27, 28]. Pavlovsky et al. [28] reported a decrease of 46–60% (from 1558 to 629–836 ng/mL) in plasma trough concentrations for imatinib 400 mg daily following SG, leading to levels below the therapeutic range during the year after, whereas the patient had adequate levels before surgery. Despite lower imatinib concentrations, complete molecular remission was maintained for 1 year after the surgery [28]. Liu and Artz [27] similarly observed a reduction in plasma trough levels of 83% (from 965 to 166 ng/mL) with imatinib 400 mg QD after a biliopancreatic diversion with duodenal switch (BDDS). The trough levels remained under the recommended threshold despite an increase to 400 mg BID. However, during a substantial weight-loss period, the levels had increased almost substantially at the same dose, which suggests a potential correlation to body weight. This is in line with our observations in case 4 in whom imatinib trough levels were measured at a significantly higher level after a period of weight loss. In the previously published case, it was suspected that post-surgery diarrhea, which is common, may influence the levels as well. MMR was not maintained following BDDS [27]. It is not clear if bariatric surgery significantly affected the efficacy of the drug as a result of reduced absorption, since response was maintained at lower trough levels post-SG in the other case [28]. Notably, BDDS is a different procedure than RYGB and SG with more substantial malabsorption.

In our case series, the measured nilotinib concentrations appeared to be highly variable following bariatric surgery and may be lower than the recommended therapeutic threshold value. Avoidance of subtherapeutic concentrations for nilotinib is of importance as an exposure-effect relation has been convincingly demonstrated for nilotinib [16]. Lower nilotinib levels following bariatric surgery are expected based on its pH-dependent solubility. Previously, lower nilotinib levels were found in 17 gastrointestinal stromal tumor (GIST) patients who had undergone gastrectomy compared with those who underwent an intestinal resection or no surgery [29]. The relative absorption was decreased by approximately 22% and 48% for respectively partial and total gastrectomy. While less influential on PK than gastrectomy, intestinal resection reduced TKI concentrations as well [29]. It must be acknowledged that gastrectomy in GIST patients may not be comparable to RYGB and SG. However, based on the physiological changes in patients with bariatric surgery, reduced levels of nilotinib may be anticipated in patients with prior bariatric surgery.

As far as we know, no previous case reports or studies on ponatinib, bosutinib, and dasatinib levels in patients with bariatric surgery have been reported. Presumed subtherapeutic levels of bosutinib were measured for cases 2 and 3. The ponatinib level measured for case 1 was in the expected range compared to a non-bariatric patient with a low dose of 15 mg OD. Before our clinical case with dasatinib treatment was referred to one of the authors’ expert clinics, trough levels of dasatinib were ordered by treating physicians intending to evaluate effective plasma levels, while for effectiveness a peak level should have been measured. Trough levels found in this patient were variable, but 3 out of 4 were below the desired limit for toxicity. The fourth was 2.6 ng/mL, only minimally above the upper limit (reference < 2.5 ng/mL).

In this case series, TKI concentrations tended to be lower in these patients who have undergone bariatric surgery when compared to data known from the general population. This is in line with changes in PK that may be induced by the anatomical and physiological changes following bariatric surgery. Anatomical changes concern a decreased gastric volume, faster gastric motility, and a reduced intestinal area for absorption depending on the type of bariatric surgery [7].

As a result of the surgery, the pH of patients with bariatric surgery can rise in the stomach pouch [5]. As nilotinib, dasatinib, ponatinib, and bosutinib all possess pH-dependent solubility, a less acidic environment can negatively affect their dissolution and absorption. This effect is probably enhanced by the use of a proton pump inhibitor. It has to be noted that the clinical significance of co-administering a TKI with proton pump inhibitors in CML treatment is still a subject of debate. Some studies suggest proton pump inhibitors reduce levels of dasatinib and nilotinib [30, 31] and may be associated with a negative impact on OS [32]. Another study indicates that the OS was not affected when the TKI was combined with proton pump inhibitors or H2 antagonists [33].

This case series is limited by the number of patients in which observations may be subject to many intraindividual factors. Second, there is substantial heterogeneity in TKI type, dosage, and clinical context. Third, drug levels were measured in routine clinical practice. There was no structured protocol for obtaining samples and therefore the available information is incomplete. As a result, there is some uncertainty about the timing of blood withdrawal as this was dependent on patient compliance with instructions and reporting. Fourth, two patients used proton pump inhibitors during the course of their disease. This may have influenced PK, although inconsistent results have been published on this issue [30, 32–34]. No other co-medication that the patients were using is known for having a clinically relevant interaction.

## Conclusions

Bariatric surgery may influence TKI levels in CML patients, leading to suboptimal response or potentially avoidable AEs. Available measured TKI levels in CML patients are scarce and highly variable. Besides molecular monitoring, we recommend using TDM as a useful tool in CML patients with obesity who undergo bariatric surgery. It should be considered to measure TKI plasma concentration pre-surgery followed by regular monitoring of TKI levels post-surgery in case of suboptimal or loss of response.

## Data Availability

The data that support the findings of this study are not openly available due to privacy or ethical restrictions. They are available from the corresponding author upon reasonable request.
